# Diagnostic and prognostic significance of serum angiopoietin-1 and -2 concentrations in patients with pulmonary hypertension

**DOI:** 10.1038/s41598-021-94907-w

**Published:** 2021-07-29

**Authors:** Noriyuki Enomoto, Seiichiro Suzuki, Hironao Hozumi, Masato Karayama, Yuzo Suzuki, Kazuki Furuhashi, Tomoyuki Fujisawa, Yutaro Nakamura, Keiichi Odagiri, Takamichi Ishikawa, Kensuke Kataoka, Yasuhiro Kondoh, Masato Maekawa, Naoki Inui, Hiroshi Watanabe, Takafumi Suda

**Affiliations:** 1 Second Division, Department of Internal Medicine, Hamamatsu University School of Medicine, Hamamatsu, Japan; 2 Health Administration Center, Hamamatsu University School of Medicine, 1-20-1 Handayama, Hamamatsu, 431-3192 Japan; 3 Department of Clinical Pharmacology and Therapeutics, Hamamatsu University School of Medicine, Hamamatsu, Japan; 4 Center for Clinical Research, Hamamatsu University Hospital, Hamamatsu, Japan; 5 Department of Pediatrics, Hamamatsu University School of Medicine, Hamamatsu, Japan; 6 Department of Respiratory Medicine and Allergy, Tosei General Hospital, Aichi, Japan; 7 Department of Laboratory Medicine, Hamamatsu University School of Medicine, Hamamatsu, Japan

**Keywords:** Biomarkers, Diagnostic markers, Prognostic markers

## Abstract

Several biomarkers for detecting pulmonary hypertension (PH) have been reported. However, these biomarkers are deemed insufficient to detect PH in its early stages. We evaluated the utility of serum angiopoietin (ANGP), a glycoprotein related to angiogenesis, as a diagnostic and prognostic biomarker of PH. Patients with PH who underwent right-heart catheterization, were retrospectively studied. Serum concentrations of ANGP-1 and ANGP-2 were measured using an enzyme-linked immunosorbent assay in patients with PH (n = 32), those with idiopathic pulmonary fibrosis (IPF) without PH (as a disease control, n = 75), and age-matched healthy controls (HC, n = 60). Nineteen patients (59.4%) with PH had World Health Organization group 3 PH. Serum ANGP-2 concentration, but not ANGP-1, in patients with PH was significantly higher compared with that in HC (p = 0.025) and in patients with IPF without PH (p = 0.008). Serum ANGP-2 concentration in patients with PH positively and significantly correlated with N-terminal pro-B-type natriuretic peptide (r = 0.769, p < 0.001), right ventricular diameter on echocardiography (r = 0.565, p = 0.035), and mean pulmonary arterial pressure (r = 0.449, p = 0.032) and pulmonary vascular resistance (r = 0.451, p = 0.031) on right-heart catheterization. ANGP-1 and ANGP-2 were expressed on lung vascular endothelial cells, as shown by immunohistochemistry. Patients with PH with higher ANGP-2 concentration (≥ 2.48 ng/mL) had significantly worse survival (p = 0.022). Higher ANGP-2 concentration was a significant worse prognostic factor (hazard ratio = 6.063, p = 0.037), while serum ANGP-1 concentration was not. In conclusion, serum ANGP-2 may be a useful diagnostic and prognostic biomarker in patients with PH, especially in patients with group 3 PH.

## Introduction

Pulmonary hypertension (PH) is a fatal disease. Early diagnosis is key to improve the prognosis of patients with PH. In addition to pulmonary arterial hypertension (PAH), several baseline diseases, such as connective tissue diseases (CTDs) or chronic lung diseases, including idiopathic pulmonary fibrosis (IPF) and chronic obstructive pulmonary disease (COPD), can cause PH^1^. Even with treatment for these diseases, concomitant PH should be detected early. The gold standard to diagnose PH is right-heart catheterization (RHC)^1^, which is relatively invasive and is not easily conducted in clinical practice. Although echocardiography is widely used to evaluate PH, the diagnostic capacity of this examination is not sufficient to diagnose PH^1^. Therefore, a novel, reliable, and easy-to-use biomarker is warranted for the diagnosis of PH.

As for peripheral blood biomarkers, although peripheral blood B type natriuretic peptide (BNP)/N-terminal pro-BNP (NT-ProBNP) has been evaluated in clinical practice^2^, these are biomarkers for heart failure following PH. In addition, several protein biomarkers such as receptor for advanced glycation end products (RAGE), insulin-like growth factor binding protein (IGFBP)-7, endostatin, collagen IV, matrix metallopeptidase (MMP)-2^2^, and MMP-7^3^, were reported to have the potential to improve early detection of PAH. To this end, further biomarkers to detect malfunction in pulmonary vessels are warranted to detect PH in its early stages.

Angiopoietin (ANGP) is a glycoprotein that plays a role in vascular development, angiogenesis, and vascular permeability. ANGP-1 and ANGP-2 competitively bind the tunica internal endothelial cell kinase-2 (Tie-2) receptor, which is primarily located on endothelial cells^4–6^. ANGP-1 is angiostatic^7^, while ANGP-2 is angioproliferative^8^ via the Tie-2 receptor. In addition, serum ANGP-2 concentration is increased in patients with acute lung injury^9^, acute respiratory distress syndrome^10^, and acute exacerbation of IPF^11,12^. In patients with PAH, ANGP-1 and ANGP-2 concentrations reportedly increased, and patients with higher ANGP-2 showed worse survival than those with lower ANGP-2^13^. However, the utility of ANGP-1 and ANGP-2 as biomarkers for other groups of PH, especially group 3 PH, has not been investigated thoroughly.

In the present study, we evaluated the capacity of serum ANGP-1 and ANGP-2 as specific diagnostic and prognostic biomarkers for pulmonary vascular abnormality in patients with PH. Furthermore, the relationships between the serum ANGP-1 and ANGP-2 concentrations and clinical parameters, especially those obtained using RHC, were examined in patients with PH.

## Results

### Clinical characteristics, World Health Organization (WHO) functional classes, clinical groups of PH, and RHC findings

Thirty-two patients who were diagnosed with PH by RHC were retrospectively enrolled. In addition to clinical data of patients with PH, clinical data of 60 age-matched healthy controls (HC) and 75 patients with IPF without PH are shown in Table 1. In patients with PH, group 3 PH occurred in 19 patients (59.4%), while group 1 PH occurred in 7 patients (21.7%). Baseline lung diseases of 19 patients with group 3 PH were IPF in 7 cases, CTD-associated interstitial lung disease (ILD) in 6 cases [systemic sclerosis (SSc)-associated ILD in 4 cases, polymyositis-associated ILD in 1 case, rheumatoid arthritis-associated ILD in 1 case], combined pulmonary fibrosis and emphysema (idiopathic interstitial pneumonia [IIPs]) in 2 cases, unclassifiable IIPs in 2 cases, COPD in 1 case, and diffuse panbronchiolitis in 1 case. Seven patients with group 1 PH had either PAH (n = 3) and CTD (n = 4). Of these with CTD, 3 had systemic lupus erythematosus (SLE) and 1 had SLE + SSc. WHO functional class II occurred in 12 patients (38%), class I occurred in 9 patients (28%), and class III occurred in 8 patients (25%). According to RHC, median value of mean pulmonary arterial pressure (PAP) was 32 mmHg and median pulmonary vascular resistance (PVR) was 5.26 Wood. The median observation period was 17.5 months in patients with PH.

**Table 1:** Patient characteristics and data of right heart catheterization in patients with PH, IPF without PH, and HC.

	PH (n = 32)	IPF (n = 75)	HC (n = 60)
Age, yo	63.5 (23, 76)	68 (47, 90)	63 (31, 72)
Sex, male/female	16 (50)/16 (50)	71 (95)/4 (5)	42 (70)/18 (30)
Observation period, months	17.5 (0, 75)	32 (0, 204)	−
**Smoking history**			
Current/ex/never	2 (6)/13 (41)/17 (53)	18 (24)/48 (64)/9 (12)	–
**Clinical groups of PH**			
1/2/3/4/5	7 (21.7)/2 (6.3)/19 (59.4)/2 (6.3)/2 (6.3)	–	–
Causes of group 1 PH	PAH 3, CTD 4 (SLE 3, SLE + SSc 1)	−	−
Causes of group 3 PH	IPF 7, CTD-ILD 6 (SSc-ILD 4, PM-ILD 1, RA-ILD 1), CPFE (IIPs) 2, UCIIP (IIPs) 2, COPD 1, DPB 1	−	−
**WHO functional classes**			
I/II/III/IV	9 (28)/12 (38)/8 (25)/3 (9)	−	−
Right heart catheterization		−	−
Mean PAP, mmHg	32 (25, 50)	−	−
PVR, wood	5.26 (0.87, 8.26)	−	−
Mean PAWP, mmHg	10 (4.9, 21)	−	−
Cardiac index, L/min/m^2^	2.74 (1.92, 5.17)	−	−

Data are presented as median (range) or n (%).

*PH* pulmonary hypertension, *PAH* pulmonary arterial hypertension, *IPF* idiopathic pulmonary fibrosis, *HC* healthy control, *CTD* connective tissue disease, *ILD* interstitial lung disease, *SLE* systemic lupus erythematosus, *SSc* systemic sclerosis, *PM* polymyositis, *RA* rheumatoid arthritis, *CPFE* combined pulmonary fibrosis with emphysema, *IIP* idiopathic interstitial pneumonia, *UCIIP* unclassifiable idiopathic interstitial pneumonia, *COPD* chronic obstructive pulmonary disease, *DPB* diffuse panbronchiolitis, *WHO* world health organization, *PAP* pulmonary arterial pressure, *PVR* pulmonary vascular resistance, *PAWP* pulmonary artery wedge pressure.

### Evaluation of serum ANGP-1 and ANGP-2 concentrations

**Figure 1:**
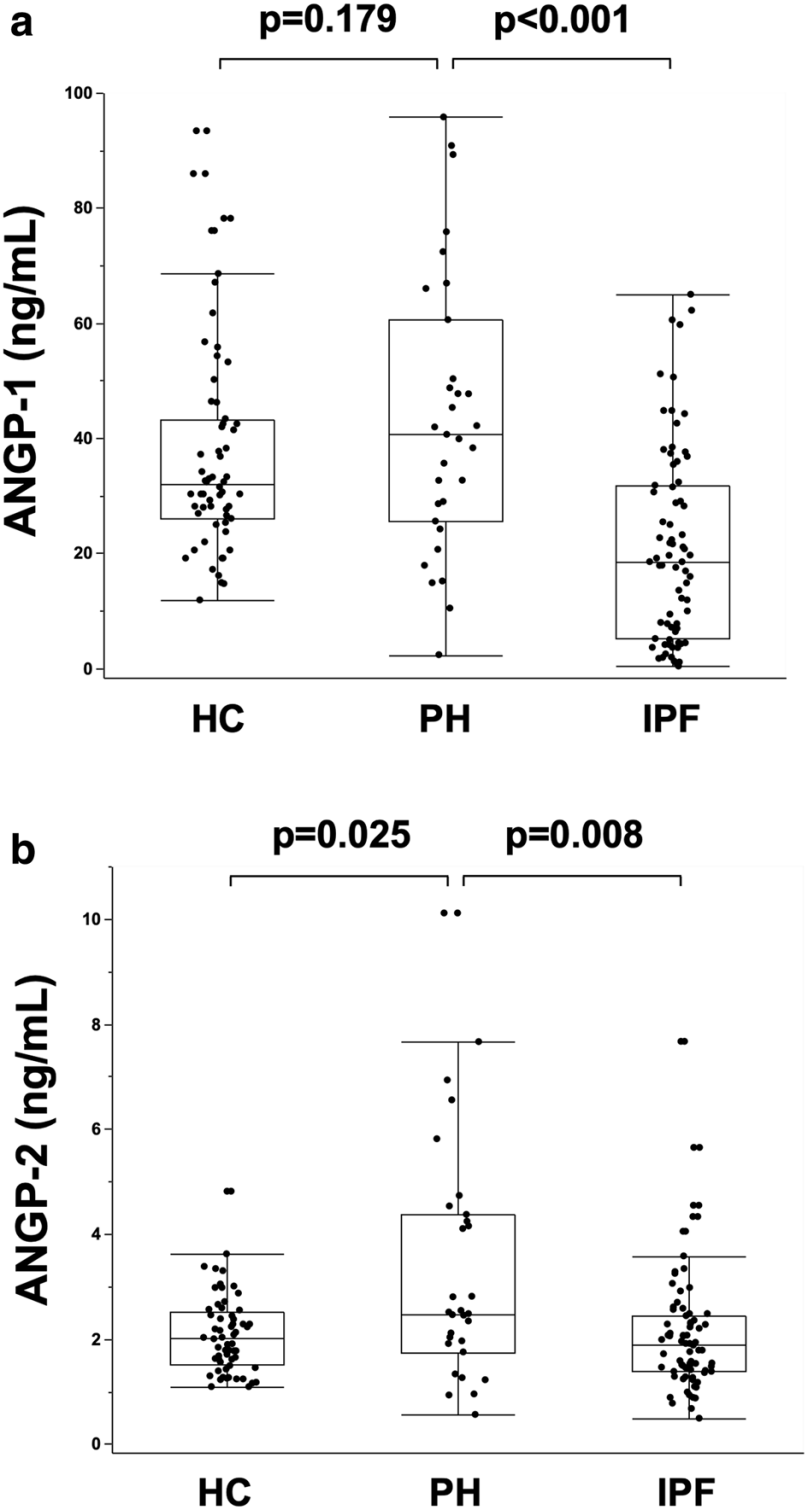
Serum concentrations of ANGP-1 and ANGP-2 in the 32 patients with PH, 60 age-matched HC, and 75 patients with IPF without PH. The serum ANGP-1 concentration in patients with PH tended to be higher but was not significantly higher compared with that in age-matched HC (**a** median, 40.6 ng/mL vs. 31.9 ng/mL [p = 0.179], respectively). The serum ANGP-1 concentration in patients with PH was significantly higher than that in patients with IPF without PH (**a** median, 40.6 ng/mL vs. 19.6 ng/mL [p < 0.001], respectively). The serum ANGP-2 concentration in patients with PH was significantly higher compared with that in age-matched HC or that in patients with IPF without PH (**b** median, 2.48 ng/mL vs. 2.01 ng/mL [p = 0.025] and 1.88 ng/mL [p = 0.008], respectively). *ANGP* angiopoietin, *PH* pulmonary hypertension, *IPF* idiopathic pulmonary fibrosis, *HC* healthy controls.

The serum concentrations of ANGP-1 and ANGP-2 in the 32 patients with PH, 60 age-matched HC, and 75 patients with IPF without PH are shown in Fig. 1. In HC, the serum ANGP-1 concentration was significantly and negatively correlated with age (Supplementary Figure E1a: r = − 0.721, p < 0.001), and the serum ANGP-2 concentration tended to be positively correlated with age (Supplementary Figure E1b: r = 0.227, p = 0.081). The serum ANGP-1 concentration in patients with PH tended to be higher but was not significantly higher compared with that in age-matched HC (Fig. 1a; median 40.6 ng/mL vs. 31.9 ng/mL [p = 0.179], respectively). The serum ANGP-1 concentration in patients with PH was significantly higher than that in patients with IPF without PH (Fig. 1a; median 40.6 ng/mL vs. 19.6 ng/mL [p < 0.001], respectively). The serum ANGP-2 concentration in patients with PH was significantly higher compared with that in age-matched HC or that in patients with IPF without PH (Fig. 1b; median 2.48 ng/mL vs. 2.01 ng/mL [p = 0.025] and 1.88 ng/mL [p = 0.008], respectively). Serum concentrations of ANGP-2 were not different between male and female in patients with PH (p = 0.163), although serum concentrations of ANGP-1 were significantly higher in female patients with PH (p < 0.001). Furthermore, in patients with PH, the serum ANGP-1 concentration was not significantly different across the WHO groups of PH (Supplementary Figure E2a). The serum ANGP-2 concentration in patients with group 3 PH was significantly higher than that in group 1 PH (Supplementary Figure E2b; median 2.81 ng/mL vs. 1.26 ng/mL, respectively; p = 0.008), although 5 of 7 patients with group 1 PH were undergoing treatment with vasodilators, while none of 19 patients with group 3 PH were treated with vasodilators at the time of ANGP measurement. Next, when limited to patients with IPF, the serum ANGP-1 and ANGP-2 concentrations could discriminate patients with PH from those without (Supplementary Figure E3a and b; p = 0.002 and p = 0.022, respectively). If the cut-off ANGP-1 concentration was defined as 40.62 ng/mL, patients with IPF and PH could be separated from those without PH with high accuracy [Supplementary Figure E3c; area under the curve (AUC) = 0.865; sensitivity, 85.7%; specificity, 88.6%]. If the cut-off ANGP-2 concentration was defined as 2.45 ng/mL, patients with IPF and PH could be separated from those without PH with moderate accuracy (Supplementary Figure E3d; AUC = 0.764; sensitivity, 71.4%; specificity, 76.0%).

### Relationships between ANGP concentrations and physiologic, echocardiographic, and RHC parameters

**Figure 2:**
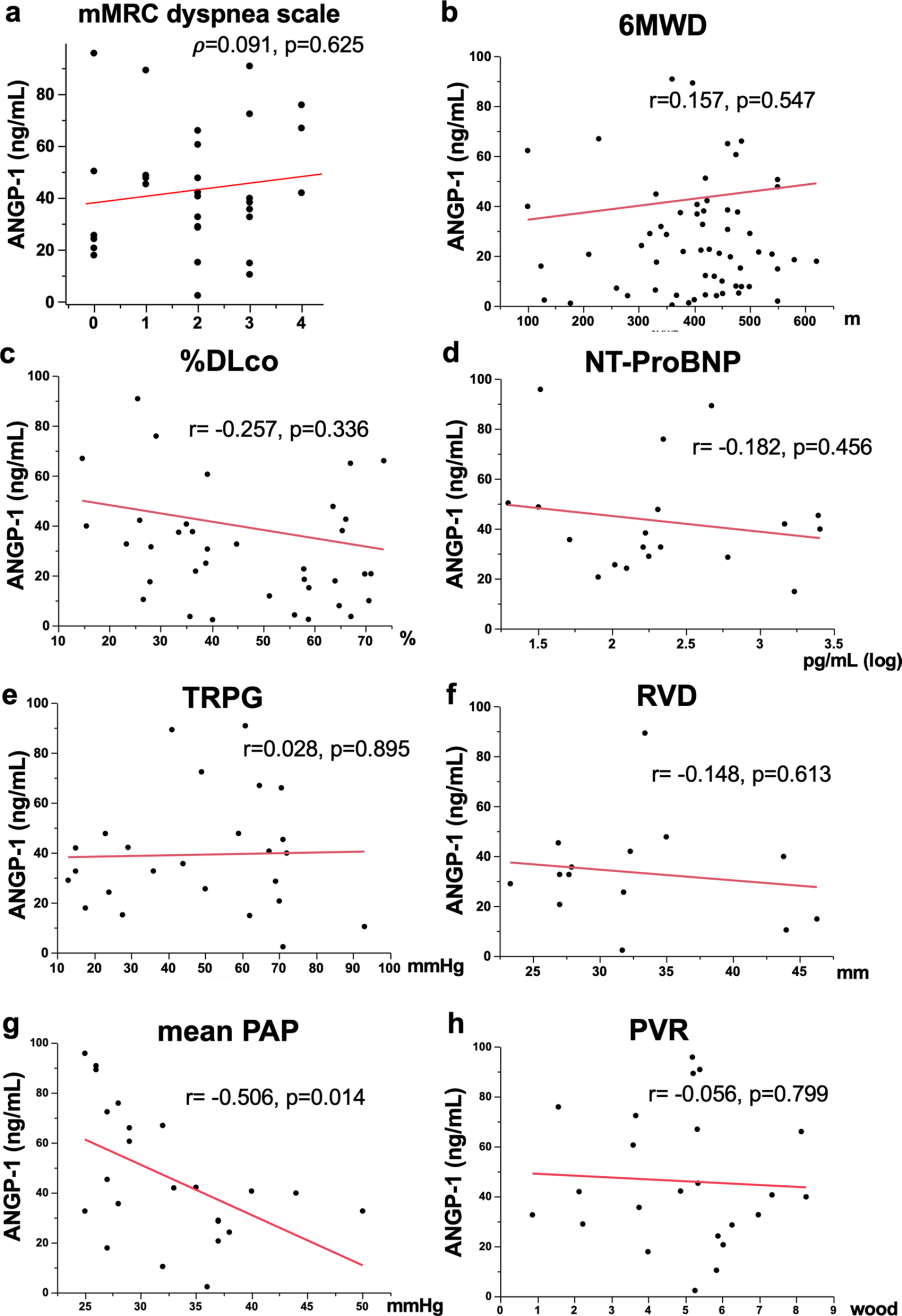
Relationships between the serum ANGP-1 concentration and several examination findings, which were simultaneously evaluated. Serum ANGP-1 concentration was not significantly correlated with mMRC dyspnea scale (**a** ρ = 0.091, p = 0.625), 6MWD (**b** r = 0.157, p = 0.547), %DLCO (**c** r = − 0.257, p = 0.336), NT-ProBNP concentration (**d** r = − 0.182, p = 0.456), or TRPG (**e** r = 0.028, p = 0.895) and RVD (**f** r = − 0.148, p = 0.613), which were evaluated by echocardiography. Serum ANGP-1 concentration was significantly and negatively correlated with mean PAP (**g** r = − 0.506, p = 0.014), although its concentration was not associated with PVR (**h** r = − 0.056, p = 0.799). *ANGP* angiopoietin, *mMRC* modified Medical Research Council, *6MWD* six-minute walk distance, *DLCO* diffusion lung capacity for carbon monoxide, *NT-Pro BNP* N-terminal pro-B-type natriuretic peptide, *TRPG* tricuspid regurgitation peak gradient, *RVD* right ventricular diameter, *PAP* pulmonary arterial pressure, *PVR* pulmonary vascular resistance.

**Figure 3:**
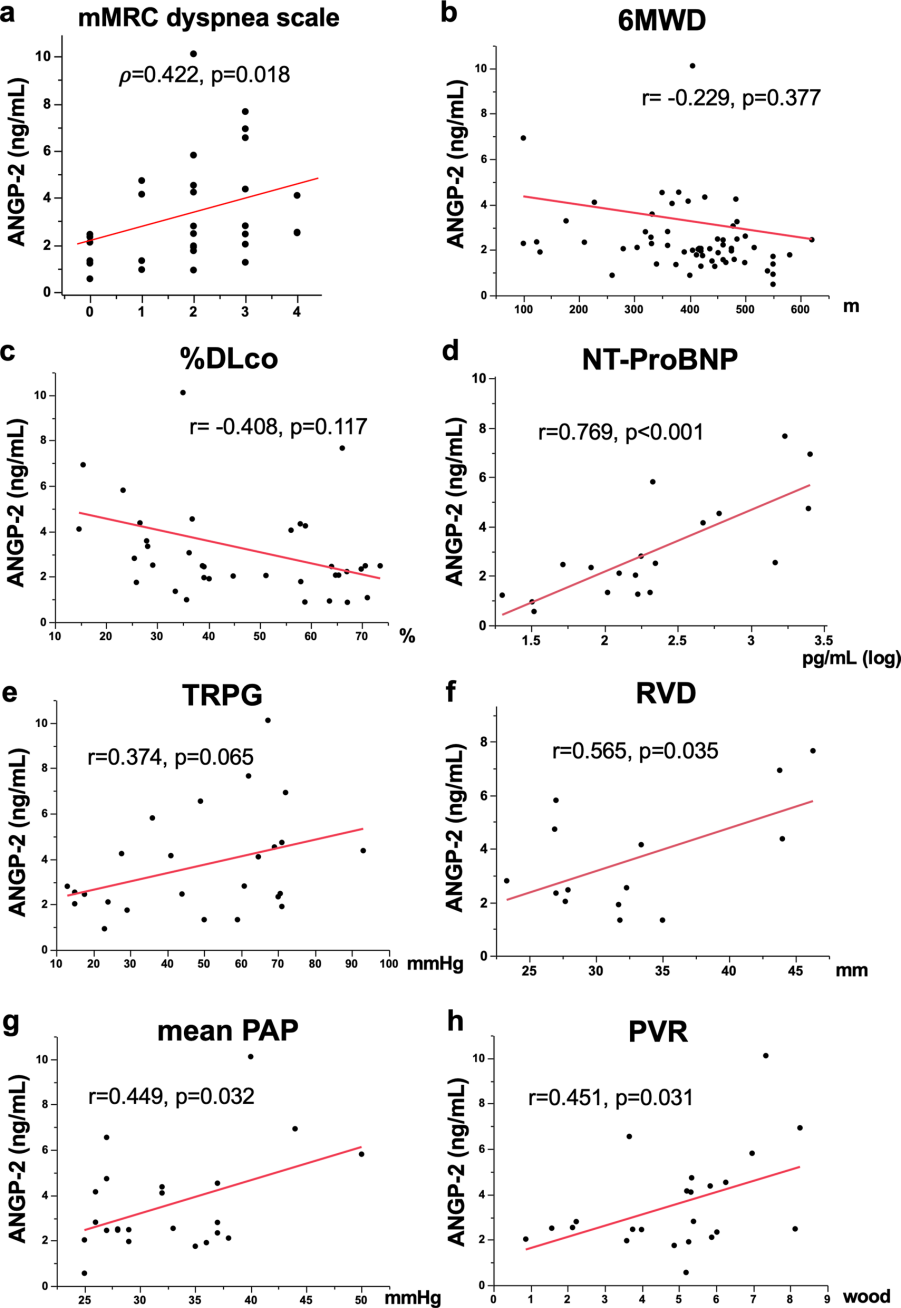
Relationships between serum ANGP-2 concentration and several examination findings, which were simultaneously evaluated. Serum ANGP-2 concentration was not associated with 6MWD (**b** r = − 0.229, p = 0.377), %DLCO (**c** r = − 0.408, p = 0.117), or TRPG (**e** r = 0.374, p = 0.065), but it was significantly and positively correlated with mMRC dyspnea scale (**a** ρ = 0.422, p = 0.018), NT-ProBNP concentration (**d** r = 0.769, p < 0.001), and RVD (**f** r = 0.565, p = 0.035). Serum ANGP-2 concentration was also significantly and positively correlated with mean PAP (**g** r = 0.449, p = 0.032) and with PVR (**h** r = 0.451, p = 0.031), which were simultaneously evaluated by RHC. *ANGP* angiopoietin, *mMRC* modified Medical Research Council, *6MWD* six-minute walk distance, *DLCO* diffusion lung capacity for carbon monoxide, *NT-Pro BNP* N-terminal pro-B-type natriuretic peptide, *TRPG* tricuspid regurgitation peak gradient, *RVD* right ventricular diameter, *PAP* pulmonary arterial pressure, *PVR* pulmonary vascular resistance.

The relationships between the serum ANGP-1 concentration and several examination findings, which were simultaneously evaluated, are shown in Fig. 2. The serum ANGP-1 concentration was not significantly correlated with modified Medical Research Council (mMRC) dyspnea scale (Fig. 2a; ρ = 0.091, p = 0.625), 6-min walk distance (6MWD; Fig. 2b; r = 0.157, p = 0.547), the percent predicted diffusion lung capacity for carbon monoxide (%DLCO; Fig. 2c; r = − 0.257, p = 0.336), NT-ProBNP concentration (Fig. 2d; r = − 0.182, p = 0.456), or tricuspid regurgitation peak gradient (TRPG, Fig. 2e; r = 0.028, p = 0.895) and right ventricular diameter (RVD, Fig. 2f; r = − 0.148, p = 0.613), which were evaluated by echocardiography. RHC was conducted in 17 of 19 patients with group 3 PH and in 2 of 7 patients with group 1 PH at the time of ANGP measurement. Serum ANGP-1 concentration was significantly and negatively correlated with mean PAP (Fig. 2g, r = − 0.506, p = 0.014), although its concentration was not associated with PVR (Fig. 2h; r = − 0.056, p = 0.799). Next, the relationship between the serum ANGP-2 concentration and these examination findings, which were simultaneously evaluated, is shown in Fig. 3. Serum ANGP-2 concentration was not associated with 6MWD (Fig. 3b; r = − 0.229, p = 0.377), %DLCO (Fig. 3c; r = − 0.408, p = 0.117), or TRPG (Fig. 3e; r = 0.374, p = 0.065); however, it was significantly and positively correlated with mMRC dyspnea scale (Fig. 3a; ρ = 0.422, p = 0.018), NT-ProBNP concentration (Fig. 3d; r = 0.769, p < 0.001), and RVD (Fig. 3f, r = 0.565, p = 0.035). Furthermore, the serum ANGP-2 concentration was also significantly and positively correlated with mean PAP (Fig. 3g; r = 0.449, p = 0.032) and with PVR (Fig. 3h; r = 0.451, p = 0.031), which were simultaneously evaluated by RHC.

### Expressions of ANGP-1 and ANGP-2 in autopsy lung specimens

**Figure 4:**
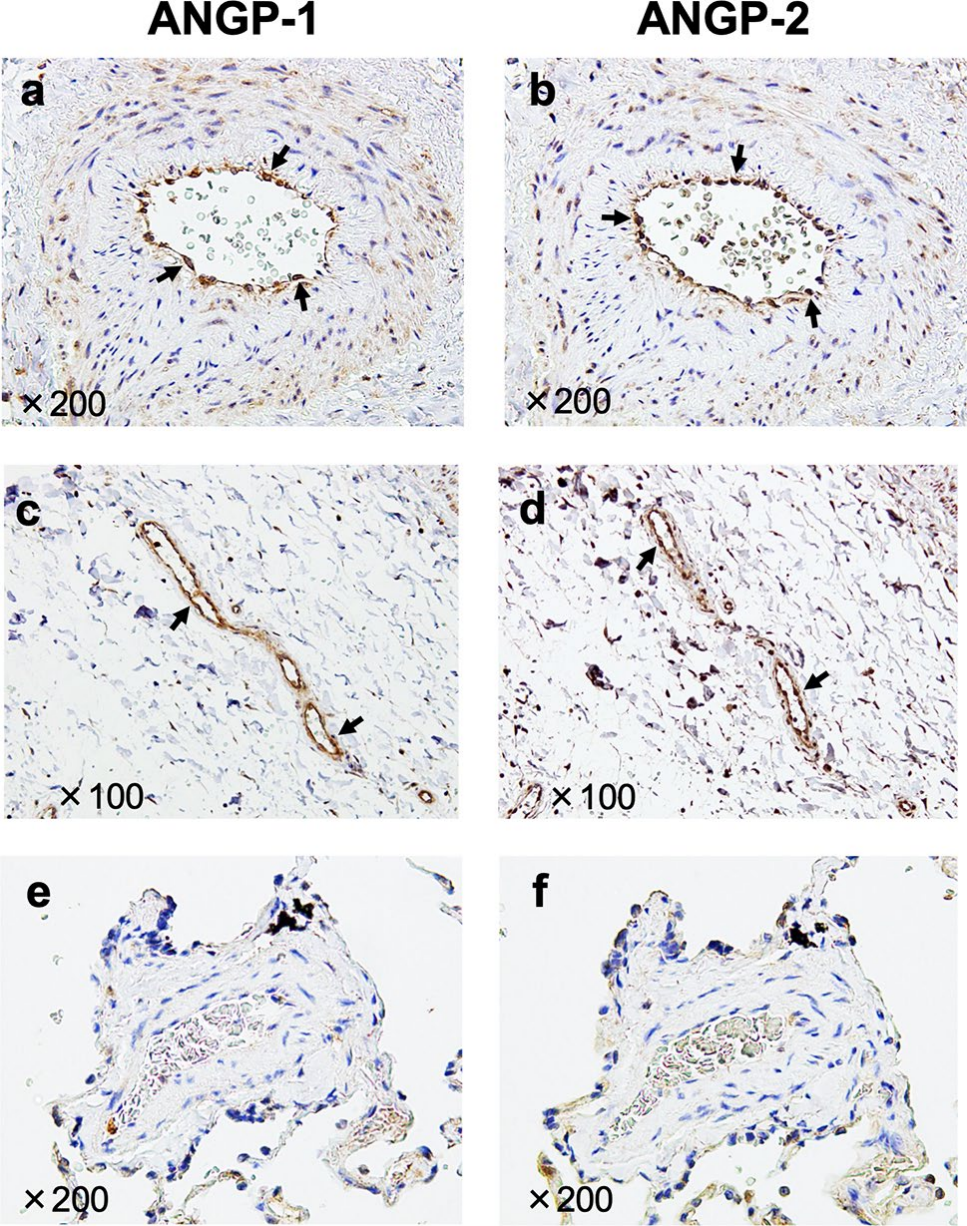
Expressions of ANGP-1 and ANGP-2 in autopsy lung specimens. The expression of ANGP-1 and ANGP-2 was evaluated with immunohistochemical staining of the lung autopsy specimens. In one patient with group 2 PH (a 23 year-old, female with patent ductus arteriosus), both ANGP-1 (**a** [×200 magnification] and **c** [×100 magnification]) and ANGP-2 (**b** [×200 magnification] and **d** [×100 magnification]) were expressed on endothelial cells of small vessels in the lungs (arrows) but not in normal lung tissue (from a 54 year-old, male with lung cancer) (**e**,**f** [×200 magnification]). *ANGP* angiopoietin, *PH* pulmonary hypertension.

The expression of ANGP-1 and ANGP-2 was evaluated by immunohistochemical staining of the lung autopsy specimens (Fig. 4). In one patient with group 2 PH (a 23 year-old, female with patent ductus arteriosus), both ANGP-1 (Fig. 4a [200× magnification] and c [100× magnification]) and ANGP-2 (Fig. 4b [200× magnification] and d [100× magnification]) were expressed on endothelial cells of small vessels in the lungs (arrows), while these were not expressed on endothelial cells of normal lung tissue (Fig. 4e,f [200× magnification], from a 54 year-old, male with lung cancer).

### Comparison between patients with low and high ANGP-2 concentration

Thirty-one patients with PH, who were followed up, were divided into two groups according to median serum ANGP-2 concentration: the ANGP-2 high group (≥ 2.48 ng/mL) and the ANGP-2 low group (< 2.48 ng/mL) (Table 2). The ANGP-2 high group included more patients with group 3 PH (p = 0.016), a higher mMRC dyspnea scale (p = 0.010), and had a higher NT-ProBNP concentration (median, 93 pg/mL vs. 608 pg/mL; p < 0.001) compared with the ANGP-2 low group. Furthermore, the ANGP-2 high group tended to have a lower %DLCO (44.8% vs. 26.6%; p = 0.072), and a higher proportion of patients treated with long-term oxygen therapy (p = 0.072) compared with the ANGP-2 low group.

**Table 2:** Comparisons between patients with PH with a high serum ANGP-2 concentration and those with a low serum ANGP-2 concentration.

	ANGP-2 low (< 2.48 ng/mL) n = 15	ANGP-2 high (≥ 2.48 ng/mL) n = 16	p value
Age, year	57 (32, 76)	67 (51, 75)	0.118
Sex, male/female	7/8	9/7	0.593
Smoking, never/ex/current	9/5/1	7/8/1	0.632
Pack-year of smoking	0 (0, 55)	20.5 (0, 126)	0.259
Emphysematous lesion on HRCT, ≥ 10%: +/−	5/10	8/8	0.346
Clinical groups of PH, 1/2/3/4/5	6/0/7/2/0	1/1/12/0/2	0.016
WHO functional classes, 1/2/3/4	6/6/3/0	3/5/5/3	0.125
mMRC dyspnea scale, 0/1/2/3/4	6/2/4/3/0	0/2/6/5/3	0.010
NT-ProBNP, pg/mL	93 (20, 205)	608 (178, 2546)	< 0.001
FVC, % pred	75 (39.4, 128)	47.1 (40.4, 110.1)	0.405
DL_CO_, % pred	44.8 (25.9, 69.8)	26.6 (14.7, 73.5)	0.072
PaO_2_ at rest, Torr	63.1 (48, 103)	58.8 (44, 101)	0.479
Distance in 6MWT, m	419 (130, 620)	360 (100, 485)	0.532
Minimum SpO_2_ in 6MWT, %	74.5 (63, 90)	79 (64, 93)	0.461
TRPG, mmHg	36.6 (15, 71)	62 (13, 93)	0.192
mean PAP, mmHg	29 (25, 38)	32 (26, 50)	0.507
PVR, wood	4.87 (0.87, 6.02)	5.37 (1.57, 8.26)	0.197
LTOT, +/−	1/14	5/11	0.072
Steroids/immunosuppressants use, +/−	6/9	6/10	0.886
PG, PDE-5 antagonist, or ERA use, +/−	7/8	4/12	0.206

Data are presented as median (range) or n.

*ANGP* angiopoietin, *HRCT* high-resolution computed tomography, *PH* pulmonary hypertension, *WHO* world health organization, *mMRC* modified medical research council, *NT-ProBNP* N-terminal pro brain natriuretic peptide, *FVC* forced vital capacity, *DLCO* diffusion lung capacity for carbon monoxide, *6MWT* 6-min walk test, *TRPG* tricuspid regurgitation pressure gradient, *PAP* pulmonary arterial pressure, *PVR* pulmonary vascular resistance, *LTOT* long term oxygen therapy, *PG* prostaglandin, *PDE* phosphodiesterase, *ERA* endothelin receptor antagonist.

### Relationship between ANGP concentrations and prognosis in patients with PH

**Figure 5:**
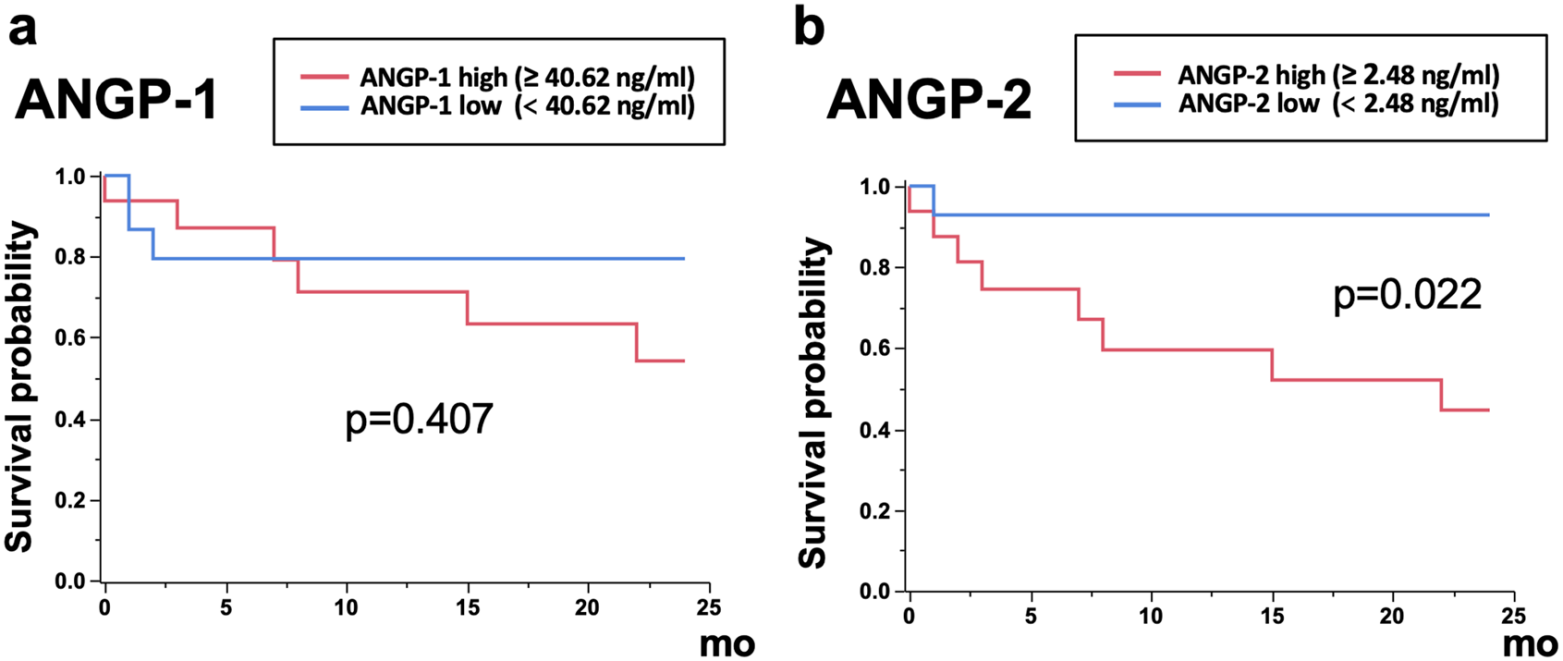
Survival curves from ANGP measurement. Thirty-one patients with PH, who were followed up, were divided into two groups according to the median serum ANGP-1 and ANGP-2 concentrations: the ANGP-1 high group (≥ 40.62 ng/mL), the ANGP-1 low group (< 40.62 ng/mL), the ANGP-2 high group (≥ 2.48 ng/mL), and the ANGP-2 low group (< 2.48 ng/mL). Kaplan–Meier curves of survival probability from the time of ANGP measurement are shown. Patients with a high ANGP-2 concentration had significantly poorer survival compared with those with a low ANGP-2 concentration (**b** log-rank, p = 0.022). The 2-year survival rate was 44.7% in the ANGP-2 high group and 92.9% in the ANGP-2 low group (**b**). Significant difference was not observed between patients with high and low ANGP-1 concentrations (**a**; p = 0.407). *ANGP* angiopoietin, *PH* pulmonary hypertension.

Thirty-one patients with PH, who were followed up, were divided into two groups according to the median serum ANGP concentrations: the ANGP-1 high group (≥ 40.62 ng/mL), the ANGP-1 low group (< 40.62 ng/mL), the ANGP-2 high group (≥ 2.48 ng/mL), and the ANGP-2 low group (< 2.48 ng/mL). The Kaplan–Meier curves of survival probability from the time of ANGP measurement are shown in Fig. 5. Patients with a high ANGP-2 concentration had significantly lower survival than those with a low ANGP-2 concentration (Fig. 5b; log-rank, p = 0.022), while a significant difference was not observed between patients with high and low ANGP-1 concentrations (Fig. 5a; log-rank, p = 0.407). The 2-year survival rate was 44.7% in the ANGP-2 high group and 92.9% in the ANGP-2 low group (Fig. 5b). When limited to patients with IPF, those with PH had significantly poorer survival compared with those without PH (Supplementary Figure E4a; log-rank, p = 0.004). Moreover, patients with IPF and PH with a high ANGP-2 concentration had significantly lower survival than those with a low ANGP-2 concentration (Supplementary Figure E4c; log-rank, p = 0.018), while a significant difference was not observed between patients with IPF and PH with high and low ANGP-1 concentrations (Supplementary Figure E4b; p = 0.330).

Next, prognostic factors from ANGP measurements were evaluated in all patients with PH. Univariable Cox proportional hazards models were used to identify factors that predict mortality in all patients with PH (Table 3). Serum ANGP-2 concentration (ANGP-2 high: hazard ratio [HR] 7.716, p = 0.015), but not the ANGP-1 concentration (p = 0.404), was a significant prognostic factor. Furthermore, WHO functional class 3–4 (HR 4.752, p = 0.017), %DLCO (HR 0.897, p = 0.036), and long-term oxygen therapy (LTOT; HR 7.135, p = 0.005) were also significant prognostic factors. Age-adjusted multivariable Cox proportional hazards models showed that serum ANGP-2 concentration was still a significant prognostic factor (Table 3; ANGP-2 high: HR 6.063, p = 0.037). Furthermore, WHO functional class 3–4 (HR 4.446, p = 0.026), %DLCO (HR 0.897, p = 0.037), and LTOT (HR 8.419, p = 0.003) still had significant prognostic value with a multivariable analysis.

**Table 3:** Cox proportional hazards models of mortality.

Variable	Univariable				Multivariable^a^			
	Hazard ratio	95% CI		p value	Hazard ratio	95% CI		p value
		Lower	Upper			Lower	Upper	
Age, year	1.029	0.974	1.112	0.352				
Sex, male	1.313	0.374	5.149	0.672				
Pack-year of smoking	1.012	0.993	1.028	0.203				
Clinical group of PH, 3 (vs. 1)	1.886	0.232	15.32	0.521				
WHO functional class, 3–4 (vs. 1–2)	4.752	1.327	18.96	0.017	4.446	1.203	18.31	0.026
FVC, % pred	0.993	0.953	1.027	0.690				
DL_CO_, % pred	0.897	0.748	0.995	0.036	0.897	0.747	0.995	0.037
PaO_2_ at rest, Torr	0.998	0.948	1.040	0.920				
Distance in 6MWT	0.997	0.990	1.004	0.423				
Minimum SpO_2_ in 6MWT	0.957	0.844	1.069	0.438				
TRPG, mmHg	1.013	0.986	1.043	0.352				
mean PAP, mmHg	0.971	0.849	1.076	0.612				
PVR, wood	0.946	0.666	1.399	0.767				
Emphysematous lesion on HRCT, ≥ 10%	2.792	0.794	10.96	0.108				
NT-ProBNP, pg/mL	1.001	0.999	1.002	0.099	1.001	0.999	1.002	0.110
Serum ANGP-1, high (vs. low)	1.781	0.468	8.458	0.404				
Serum ANGP-2, high (vs. low)	7.716	1.414	143.2	0.015	6.063	1.099	112.9	0.037
LTOT, +	7.135	1.853	29.32	0.005	8.419	2.143	35.24	0.003
Steroids/immunosuppressants use, +/−	2.292	0.634	8.286	0.198				
PG, PDE-5 antagonist, or ERA use, +/−	0.517	0.078	2.073	0.376				

*PH* pulmonary hypertension, *WHO* world health organization, *FVC* forced vital capacity, *DLCO* diffusion lung capacity for carbon monoxide, *6MWT* 6-min walk test, *TRPG* tricuspid regurgitation pressure gradient, *PAP* pulmonary arterial pressure, *PVR* pulmonary vascular resistance, *HRCT* high-resolution computed tomography, *NT-ProBNP* N-terminal pro brain natriuretic peptide, *ANGP* angiopoietin, *LTOT* long term oxygen therapy, *PG* prostaglandin, *PDE* phosphodiesterase, *ERA* endothelin receptor antagonist.

^a^Age-adjusted multivariable models.

## Discussion

In the present study, 32 patients with PH, mainly group 3 PH (19 patients [59.4%]), and 75 patients with IPF without PH were retrospectively studied. Serum ANGP-2 concentration, but not ANGP-1, in patients with PH was significantly higher compared with that in age-matched HC, and that in IPF patients without PH. Serum ANGP-2 concentration in patients with PH positively and significantly correlated with mMRC dyspnea scale, NT-ProBNP/BNP concentration, RVD on echocardiography, and mean PAP and PVR on RHC. Patients with PH with a higher serum ANGP-2 concentration showed significantly worse survival. A high ANGP-2 concentration was a predictor of a poor prognosis, while serum ANGP-1 concentration was not. To our knowledge, this is the first study that showed the significance of ANGP-2 as a diagnostic and prognostic biomarker for mainly group 3 PH.

The gold standard of PH diagnosis is RHC^1^, although RHC is relatively invasive and is not easy to conduct in clinical practice. NT-ProBNP/BNP, %DLCO, and echocardiography are less invasive, but their diagnostic and prognostic significance are not sufficient in patients with PH. Therefore, a novel and reliable biomarker that is easy to measure is desired for PH. Previous studies have reported that plasma endothelin-1 and cyclic guanosine monophosphate (cGMP)/NT-proBNP ratio were related to 6MWD^14^, and increased serum interleukin (IL)-6/IL-8/IL-10/IL-12 predicted worse survival^15^ in patients with PAH. In the current study, although cGMP and cytokines were not evaluated, serum ANGP-2 was identified as a useful biomarker for the diagnosis of PH. Moreover, ANGP-2 predicted survival in patients with PH.

ANGP-1 and ANGP-2 play a role in vascular development, angiogenesis, and vascular permeability. ANGP-1 and ANGP-2 competitively bind the Tie-2 receptor, which is primarily found on endothelial cells^4–6,16^. Binding of ANGP-1 to Tie-2 on endothelial cells promotes vascular integrity and leads to an angiostatic “non-leaky” condition^7^, while ANGP-2 binding to Tie-2 promotes angiogenesis and vascular permeability^17^. In the current study, serum ANGP-2 concentration in patients with PH was significantly higher compared with that in patients with IPF without PH and that in age-matched HC. In the early stage of group 3 PH, hypoxic vasoconstriction and loss of vascular bed lead to the redistribution of pulmonary circulation^18^. In such a circumstance, the recruitment capacity and distensibility of pulmonary vessels play a role to keep a functional pulmonary circulation and gas-exchange^19,20^. ANGP-2 is mainly produced from endothelial cells^21^, and ANGP-2-induced angiogenesis may keep such a functional gas-exchange at the distended pulmonary vessels. In patients with PAH, ANGP-2 concentration reportedly showed positive correlation with PVR, and an elevated ANGP-2 concentration was associated with worse survival^13^. According to our study, patients with PH with a higher serum ANGP-2 concentration showed significantly worse survival, and a higher ANGP-2 concentration predicted a worse prognosis. A previous study reported that hypoxia, vascular endothelial growth factor, and basic fibroblast growth factor increased ANGP-2 release from endothelial cells^21^. Furthermore, ANGP-2 was expressed only at sites of vascular remodeling in a rodent model^8^. Therefore, excessive production of ANGP-2, which promotes angiogenesis, may be related to PH disease progression and may be a novel therapeutic target in patients with PH.

In the present study, patients with PH mainly presented with group 3 PH (59.4%). Fifteen percent of patients with IIP and 27% of those with combined pulmonary fibrosis and emphysema had group 3 PH^22^. Furthermore, in terms of IPF, 14% of patients with IPF with a mild-to-moderate restriction in lung volume^23^ and 31.6% of patients with advanced IPF had PH^24^. Many previous studies focused on group 1 PH, especially PAH. Major differences between group 1 PH and group 3 PH are that group 3 PH demonstrates poorer RV function, a lower mean PAP and PVR, and worse survival^25^. Although vasodilator treatment is less efficient or worsens the respiratory condition in patients with group 3 PH^25^, a mean PAP of ≥ 35 mmHg (severe PH) warrants consideration of vasodilator therapy. Moreover, a recent study showed that inhalational treprostinil in patients with group 3 PH could increase 6MWD and decrease NT-proBNP concentration^26^. In the present study, serum concentrations of ANGP-2 were significantly higher than in age-matched HC or patients with IPF (Fig. 1a). Therefore, the serum concentration of ANGP-2 may be a useful diagnostic biomarker in patients with PH, and the regular measurement of ANGP-2, in combination with other biomarkers, in patients with pulmonary diseases may be practical for the early detection of PH. Furthermore, serum ANGP-2 concentration was positively and significantly correlated with mean PAP and PVR. Therefore, less invasive biomarkers, such as serum ANGP-2, may be useful for clinical decision making in terms of PH treatment in patients with group 3 PH.

The present study has several limitations. First, this study adopted a retrospective design, and the number of patients enrolled was relatively small, which was also more group 3 PH predominant compared to the common distribution of PH prevalence. In addition, immunohistochemical analysis was conducted in only two due to the limited number of patients whose lung autopsy specimens were available. Second, longitudinal ANGP values were not evaluated. Third, treatments were not uniform across PH groups when serum ANGPs were measured, and may have affected serum concentrations of ANGPs. Therefore, larger prospective studies are needed to confirm the significance of ANGPs in patients with PH.

In conclusion, the capacity of serum ANGP-1 and ANGP-2 as diagnostic and prognostic markers was retrospectively evaluated in patients with PH. Serum ANGP-2 concentration, but not ANGP-1, in patients with PH was significantly higher compared with that of age-matched HC and that of patients with IPF without PH. Serum ANGP-2 concentration in patients with PH positively and significantly correlated with mean PAP and PVR on RHC. Patients with PH with a higher serum ANGP-2 concentration showed significantly worse survival. A higher ANGP-2 concentration was a significant predictor of a worse prognosis. Collectively, these results suggest that serum ANGP-2 is easy to evaluate and may be useful as a diagnostic and prognostic biomarker for PH. Future studies should prospectively confirm the significance of ANGP-2 as a practicable biomarker in patients with PH.

## Methods

### Study design and patients

Thirty-two patients, who were diagnosed with PH from 2000 to 2020, were retrospectively studied. All patients were diagnosed with PH by RHC using a mean PAP of ≥ 25 mmHg. Seventy-five patients with IPF without PH were also studied. These patients met the IPF consensus criteria of the American Thoracic Society/European Respiratory Society/Japanese Respiratory Society/Latin American Thoracic Association^27^. All procedures in this study were performed in accordance with the study protocol and the 1964 Helsinki Declaration, as amended. The need for patient approval and informed consent was waived by Ethics Committee of Hamamatsu University School of Medicine due to the retrospective nature of the study. However, informed consent was obtained from all patients still visiting our clinics. The study protocol was approved by the Ethics Committee of Hamamatsu University School of Medicine (approval number: 17-232).

### Data collection

Clinical, laboratory, and physiological data were obtained from medical records, which included FVC, DLCO, and partial pressure of oxygen at rest, minimum oxygen saturation, and walk-distance during the 6MWT. The extent of pulmonary emphysematous lesions on high-resolution computed tomography was evaluated by two observers. Echocardiography and RHC findings were also obtained from medical records. These data, which were measured on the date closest to the date on which the serum ANGP was evaluated, were used. Survival was analyzed from the date on which serum ANGP was measured.

### Measurement of serum ANGP

Blood samples were collected from enrolled patients with PH. Serum concentrations of ANGP-1 and ANGP-2 were measured using an enzyme-linked immunosorbent assay (R&D Systems, Inc., Minneapolis, MN, USA). ANGP concentrations were compared with those of patients with IPF without PH (as a disease control) and age-matched HC.

### Immunohistochemical staining of ANGPs

ANGPs were immunohistochemically stained in autopsy specimens. Briefly, deparaffinized sections were steeped in 0.3% hydrogen peroxide to inactivate endogenous peroxidase activity and then blocked with 10% normal goat serum. Sections were incubated with rabbit polyclonal antibody against ANGP-1 and ANGP-2 (anti-ANGPT1 or anti-ANGPT2 (C-term), Sigma-Aldrich, Saint Louis, MO, USA). After rinsing with phosphate-buffered saline, sections were incubated with biotin-conjugated goat anti-rabbit immunoglobulin G polyclonal antibody (Nichirey, Tokyo, Japan). Sections were then incubated with streptoavidin-peroxidase complex (Nichirey, Tokyo, Japan). The antigen–antibody complex was visualized with 3,3′-diaminobenzidine (DAB; Nichirey, Tokyo, Japan) and counterstained with hematoxylin.

### Statistical analysis

Statistical analyses were performed using JMP 13.1.0 (SAS Institute Inc., Cary, NC, USA). Categorical data were compared using the χ^2^ test or Fisher’s exact probability test for independence. Continuous data were analyzed using the Wilcoxon rank-sum test. Relationships between the serum ANGP concentrations and serial data were analyzed using Pearson’s correlation coefficient. Relationships between the serum ANGP concentrations and categorical data were analyzed using Spearman’s rank correlation coefficient. Overall survival of patient groups was estimated using the Kaplan–Meier method and Kaplan–Meier curves were compared using the log-rank test. The relationships between variables and mortality were evaluated using the Cox proportional hazards regression analysis. All tests were two-sided, and a p value of < 0.05 was considered statistically significant.

## Supplementary Information

Supplementary Information 1.

Supplementary Information 2.

Supplementary Information 3.

Supplementary Information 4.

Supplementary Information 5.

## Data Availability

The data that support the findings of this study are available from the corresponding author, NE, upon reasonable request.
