# Supplementary material for: Diagnostic and prognostic significance of serum angiopoietin-1 and -2 concentrations in patients with pulmonary hypertension
Source: Sci Rep. 2021 Jul 29;11:15502. doi: 10.1038/s41598-021-94907-w (PMC8322335; doi:10.1038/s41598-021-94907-w)
Supplement: Supplementary file 1 — Supplementary Information 1. [file 41598_2021_94907_MOESM1_ESM.docx]

**Supplementary Figure E1.** In HC, the serum ANGP-1 concentration was significantly and negatively correlated with age (a: r= -0.721, p<0.001). The serum ANGP-2 concentration tended to be positively correlated with age (b: r= 0.227, p=0.081). Abbreviations: HC: healthy controls, ANGP: angiopoietin.

**Supplementary Figure E2.** Serum concentrations of ANGP-1 and ANGP-2 in each WHO group of PH. In patients with PH, the serum ANGP-1 concentration was not significantly different across the WHO groups of PH (a). Serum ANGP-2 concentration in patients with group 3 PH was significantly higher compared with that in patients with group 1 PH (b; median, 2.81 ng/mL vs. 1.26 ng/mL, respectively; p=0.008). Abbreviations: ANGP: angiopoietin, WHO: World Health Organization, PH: pulmonary hypertension.

**Supplementary Figure E3.** Serum ANGP-1 and ANGP-2 concentrations in patients with IPF, and receiver operating characteristic curve for discrimination between IPF patients with PH and those without PH. Serum ANGP-1 and ANGP-2 concentrations could discriminate patients with IPF with PH from those without PH (a and b; p=0.002 and p=0.022, respectively). If the cut-off ANGP-1 concentration was defined as 40.62 ng/mL, patients with IPF with PH could be separated from those without PH with high accuracy (c; AUC=0.865; sensitivity, 85.7%; specificity, 88.6%). If the cut-off ANGP-2 concentration was defined as 2.45 ng/mL, patients with IPF with PH could be separated from those without PH with moderate accuracy (d; AUC=0.764; sensitivity, 71.4%; specificity, 76.0%). Abbreviations: ANGP: angiopoietin, PH: pulmonary hypertension, IPF: idiopathic pulmonary fibrosis, AUC: area under the curve.

**Supplementary Figure E4.** Survival curves from ANGP measurement in patients with IPF. Patients with IPF with PH had significantly poorer survival compared with those without PH (a; log-rank p=0.004). Moreover, patients with IPF and PH with a high ANGP-2 concentration had significantly poorer survival compared with patients with a low ANGP-2 concentration (c; log-rank p=0.018), while a significant difference was not observed between patients with IPF and PH with high and low ANGP-1 (b; p=0.330). Abbreviations: ANGP: angiopoietin, PH: pulmonary hypertension, IPF: idiopathic pulmonary fibrosis.
