# Supplementary figures and images for: Diagnostic and prognostic significance of serum angiopoietin-1 and -2 concentrations in patients with pulmonary hypertension
Source: Sci Rep. 2021 Jul 29;11:15502. doi: 10.1038/s41598-021-94907-w (PMC8322335; doi:10.1038/s41598-021-94907-w)

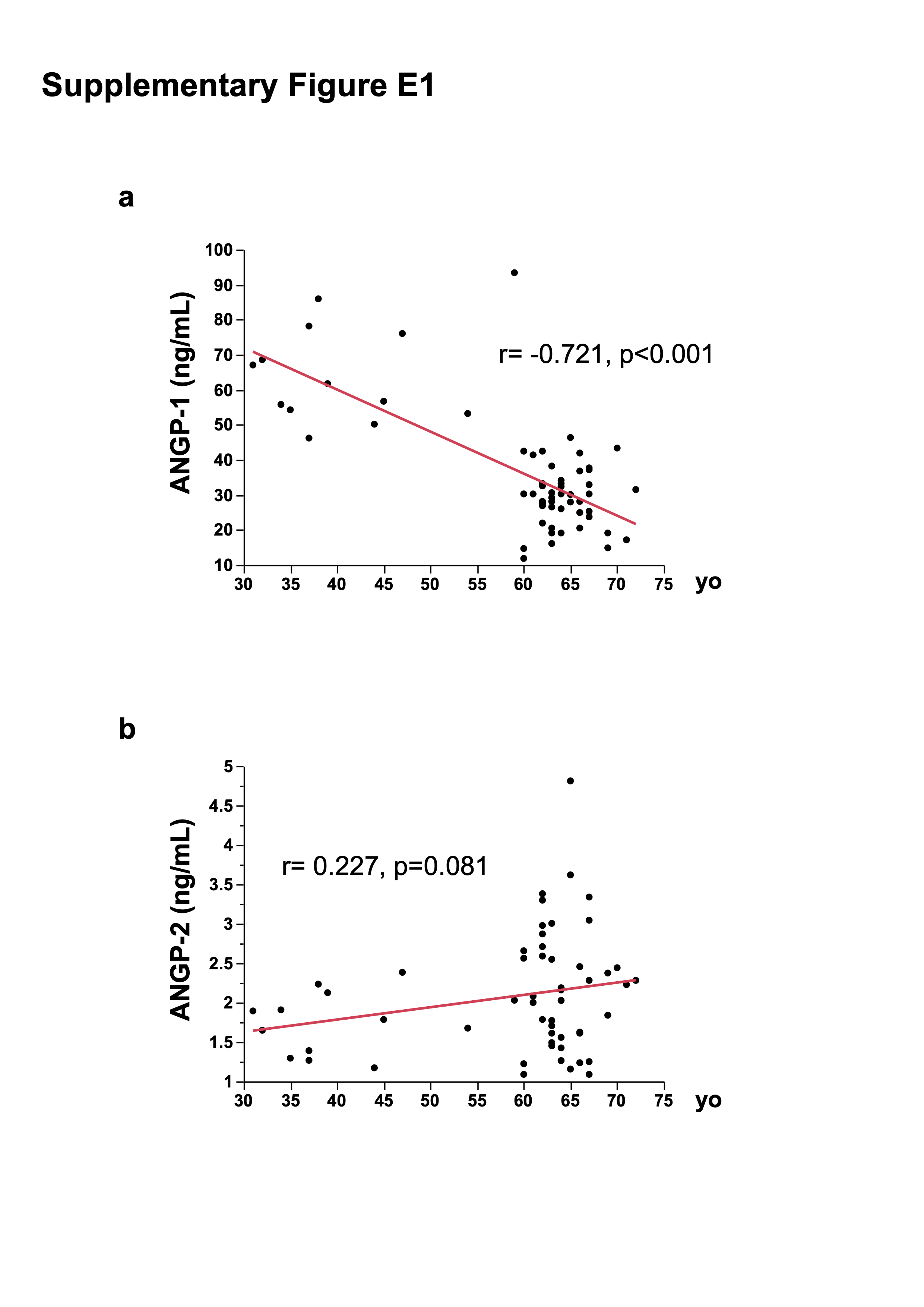

Supplement: Supplementary file 2 — Supplementary Information 2. [file 41598_2021_94907_MOESM2_ESM.tiff]

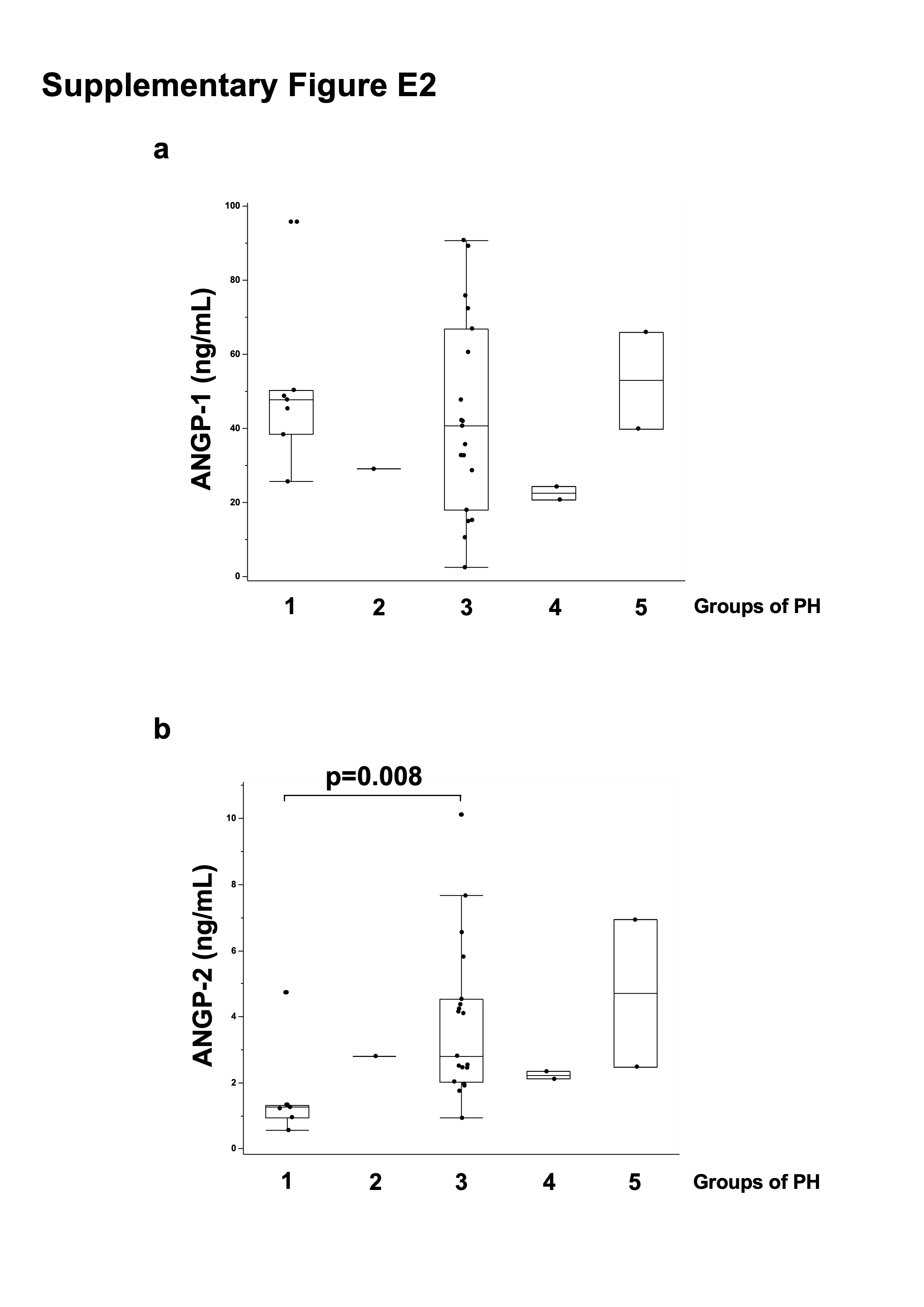

Supplement: Supplementary file 3 — Supplementary Information 3. [file 41598_2021_94907_MOESM3_ESM.tiff]

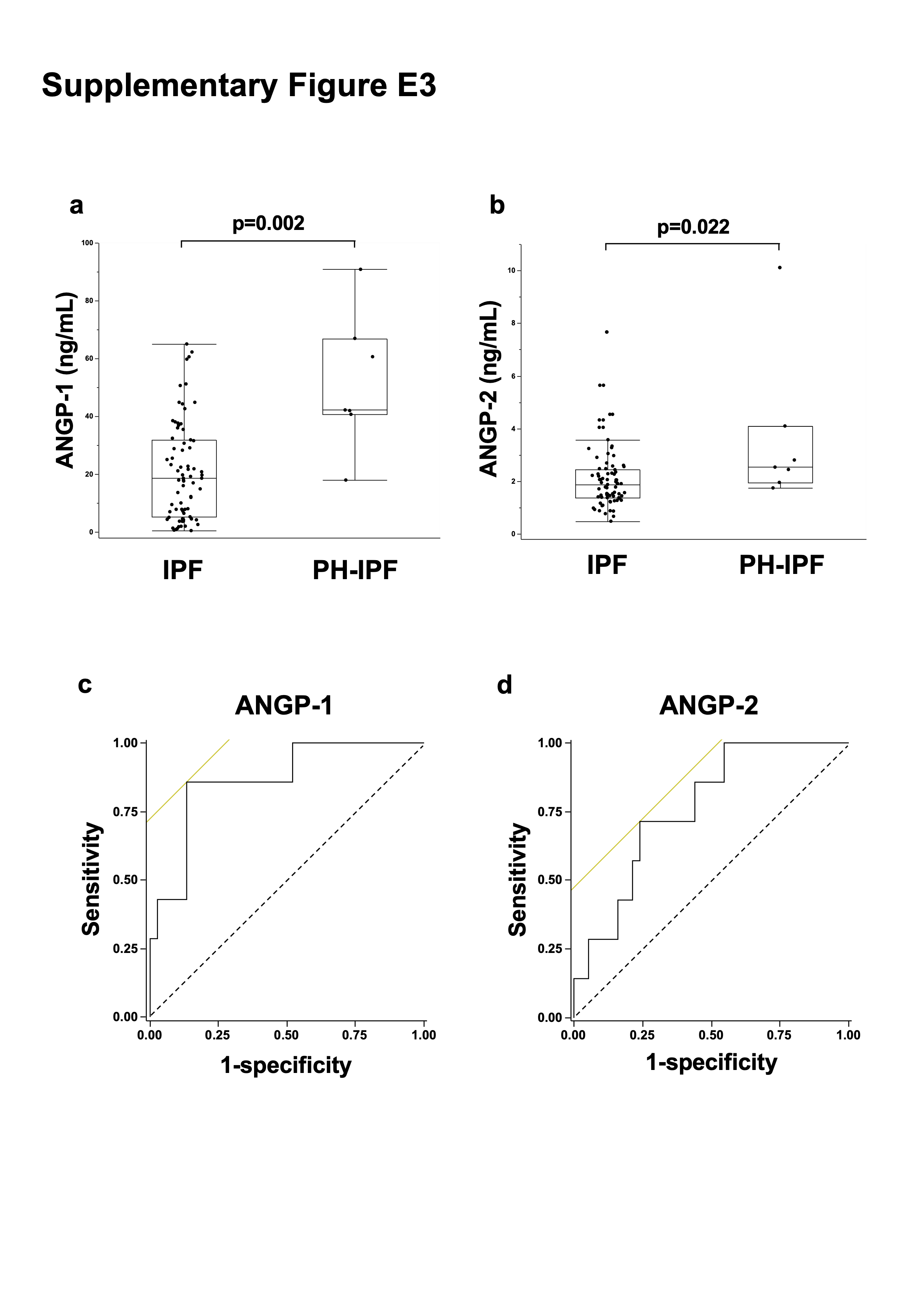

Supplement: Supplementary file 4 — Supplementary Information 4. [file 41598_2021_94907_MOESM4_ESM.tiff]

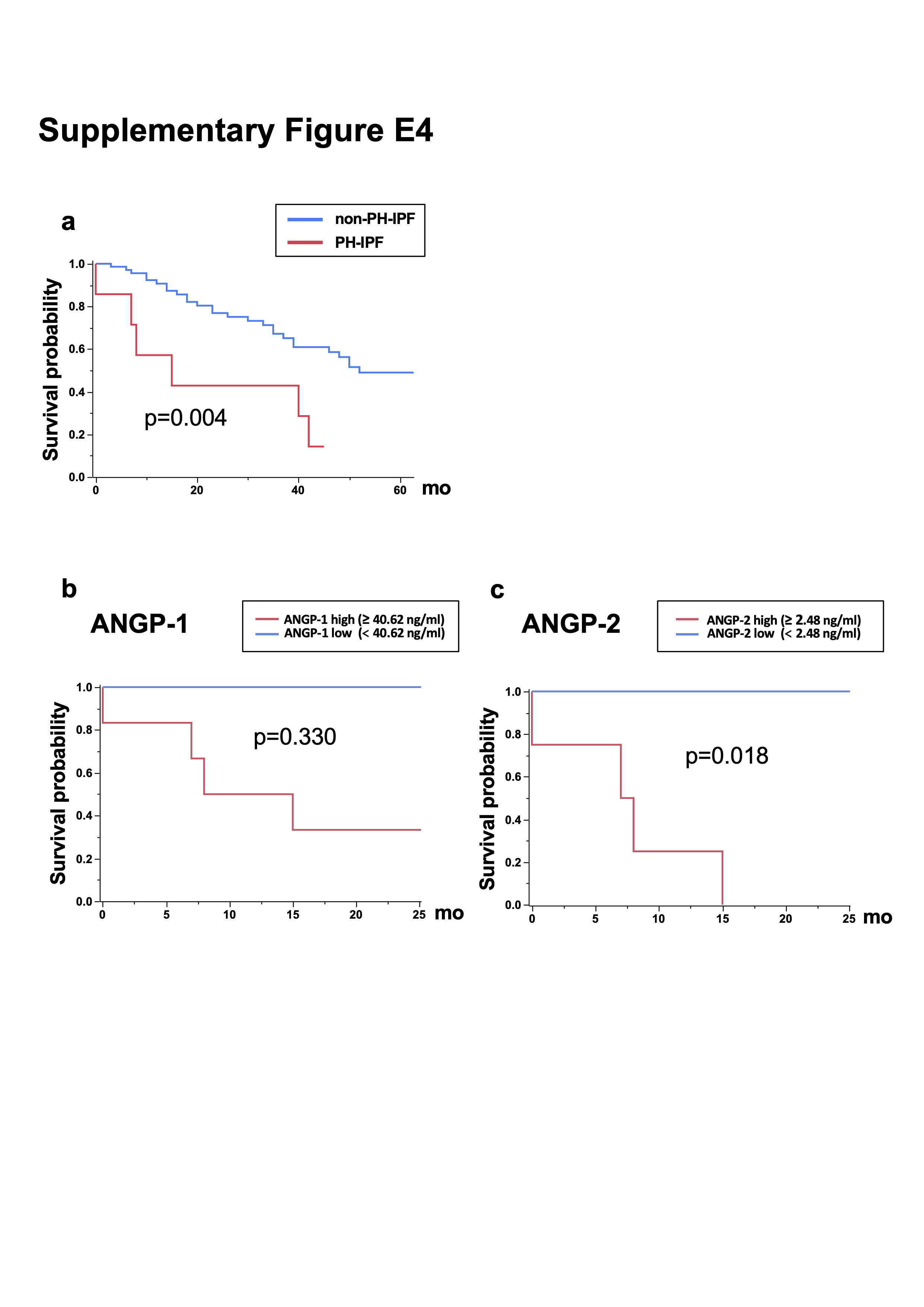

Supplement: Supplementary file 5 — Supplementary Information 5. [file 41598_2021_94907_MOESM5_ESM.tiff]
